# The Potential of Specialized Media in Public Health: Analysis of Health-Related Content in Sports Newspapers

**DOI:** 10.3390/ijerph16071202

**Published:** 2019-04-03

**Authors:** Antonio Lopez-Villegas, Daniel Catalan-Matamoros

**Affiliations:** 1 Research Group of Social Involvement of Critical and Emergency Medicine CTS-609, Hospital de Poniente, 04700 Almeria, Spain; antoniolopezvillegas@andaluciajunta.es; 2 Institute of Clinical Medicine, University of Tromsø, 9019 Tromsø, Norway; 3 Department of Journalism and Communication, University Carlos III of Madrid, 28903 Getafe, Spain; 4 Research Group of Health Sciences CTS-451, University of Almeria, 04120 Almería, Spain

**Keywords:** health communication, newspapers, physical activity, public health, Spain, sports

## Abstract

Sports-specialized newspapers are one of the print media with the highest number of readers in Spain. However, little is known about the health coverage in this type of print press. The aim of the study was to analyze any health-related material in sports newspaper coverage and describe the main characteristics. This is an observational and cross-sectional study, performed in relation to the three most read daily Spanish sports newspapers (MARCA, AS, SPORT). A descriptive analysis was conducted to assess the health-related materials selected after a careful search over a period of 30 days. During this time, a total of 815 units of analysis were identified. On average, 14.79% (*n* = 645 pages) of the full content (*n* = 4362) included health-related material. The Liga BBVA section was the most frequent to contain health-related content by a significant margin (*p* = 0.01). The main covered topics were injuries to soccer players (52%), doping (21%), and other diseases in athletes or their relatives (8.6%) with no significant differences (*p* = 0.10). Photographs (87.4%) were the most frequent visual material used in the health content, followed by infographics (12.6%). Press releases were the most frequent source of information (58%). Spanish sports newspapers include a high proportion of health-related material, especially in terms of providing detailed descriptions of athletes’ sport injuries, mainly related to soccer.

## 1. Introduction

Sport is a large, fast-growing sector of the economy and is responsible for around 2% of global Gross Domestic Product (GDP). It also makes an important contribution to growth and jobs, with employment effects exceeding average growth rates [1]. The sports sector employs 15 million people or 5.4% of the labor force [2]. In addition, major sporting events and competitions have considerable potential to generate increased tourism in Europe [3].

The relationship between the sports sector and the media is critically important. Since the mass media, especially television, create big audiences, they have become interesting partners with sports for economic and political purposes. In Europe, television broadcasting rights are the primary income source for professional sport [2]. Diverse sporting events including the Olympic Games, athletics world championships, major professional soccer championships and leagues, tennis grand slams, Formula 1 races, etc. are an important source of content for the sports media. 

The sports press has not stopped growing since William Randolph Hearst (1863–1951), the owner of the *New York Journal*, published the first sports news in a general newspaper in 1895. In fact, sports coverage immediately became one of his favorite and most successful strategies [4]. Sport is currently one of the main leisure activities, as a result of a process of development and dissemination throughout the 20th century, motivated by many key factors, including the mass media. 

### 1.1. The Sports Press

The sports press is no longer limited to narrating sporting events. Consumers of sports news demand access to other aspects of sport and athletes, including social, economic, and health issues. Because sports newspapers receive a high number of readers in Spain, they have enormous potential as a tool for health promotion and disease prevention [5]. Since there is an increasing interest in health issues in the mass media [6,7,8], the number of health-related materials published are also increasing in the sports newspapers, which may thus be considered an important medium to promote education and public health through their contents.

Spain holds some popular sporting events that are followed not only by Spaniards but also internationally. For example, millions of fans around the world follow the premier soccer league “La Liga.” “Real Madrid F.C.” and “Barcelona F.C.” are recognized references in world soccer, and in sport generally. In addition, Spanish athletes excel in many sports and are in the world elite, like Mario Mola in the triathlon [9] or Rafael Nadal in tennis [10]. In Spain, the sports press is booming and receives the primary positions in a number of newspaper as regards numbers of readers. According to a national media study in Spain [11], “Marca,” the most popular daily sports newspaper, was read by almost twice as many people than the first general daily newspaper “El Pais” (1.7 vs. 1.0, respectively). 

### 1.2. Justification and Objective

Previous studies have analyzed all health-related materials in general newspapers [8,12]. Others have analyzed specific sports [13,14,15,16], organic diseases [17,18,19,20,21,22,23,24] and/or psychological illness [25,26,27,28,29,30]. A similar previous study was published [18]; however, the analysis only focused on athletes’ medical information disclosed by English newspapers. To our knowledge, there are no studies analyzing any health-related materials in sports newspapers. Therefore, the objective of this study was to carry out a descriptive analysis of the health-related material published in Spanish sports newspapers and to identify the challenges and opportunities faced by this type of specialist journalism.

## 2. Material and Methods

### 2.1. Study Design 

This study followed an observational and cross-sectional design. It consists in a structured observation which selected the health news included in our sample of sports newspapers. The sample consisted of 90 print editions of the three most popular Spanish sports newspapers: MARCA, AS, and SPORT, with 1.7, 0.8, and 0.4 million daily readers, respectively [11]. Consecutive print editions were collected over a period of 30 days (one full month). The study period was selected following a randomized system using the online service of ‘randomized.org,’ which followed this process: we included the 12 months and the last 10 years (2008–2017). Finally, the month and year were randomly selected. The final period of study was ‘December 2011.’ The unit of analysis was the health-related material (texts, images, illustrations, etc.) that appeared in the selected sports newspapers during the sampling period [31]. 

### 2.2. Study Protocol

Once the health-related material had been extracted, it was analyzed by the research group. A template was designed to collect the following variables: (1) identified items: newspaper name, publication date, size, and page number; and (2) descriptive items: section, genre, subgenre, page, author (name and affiliation), topic, illustrations, and sources of information. The variables selected for analysis were the same as those used in similar previous analyses of the general print press [8,32]. Data collection was conducted from records and based on an observational model that consists of a careful review of these sports newspapers to pick out everything related to health.

### 2.3. Variables Coded, Instrument Development, Coder Training, and Intercoder Reliability

The two authors (ALV & DCM) tested an initial draft of the coding instrument informally by independently coding five print editions of the sports newspapers selected, three from within the sample and two that were published a week prior to those in the sample. On the basis of this test, problems and disagreements related with coding were discussed and the form was revised. This protocol was repeated four times until the instrument was considered reliable, then a reliability pilot test was formally conducted using the following methods. To establish the intercoder reliability, both authors coded 60 (66.67%) of the sports newspaper units. In addition, each author coded half of the remaining 30 sports newspapers. To build the final database, the sports newspapers used in the reliability analysis were divided randomly into two different groups and the decisions of each coder were randomly selected. Percent agreement, Scott’s pi, Cohen’s kappa, and Krippendorff’s alpha were used to assess intercoder reliability for each variable [33]. ReCal (“Reliability Calculator”) software was used to calculate percent agreement, Scott’s pi, Cohen’s kappa, and nominal Krippendorff’s alpha [34,35]. Holsti’s method is not included because it is identical to Scott’s pi in the case of two coders evaluating the same variable. Besides, to consider the coding of a variable reliable, either a Krippendorff’s alpha ≥0.70, or a percent agreement of ≥0.90 is needed. 

### 2.4. Statistical Analysis

Characteristics and potential differences between groups were compared using a difference in means test for continuous variables and a difference in proportions test (binomial method) or chi-square test (replaced by the Fisher exact test for cells with *n* < 5 cases) for qualitative variables. Differences between groups in the pre-specified endpoints were also assessed using the difference in means or proportions tests. Results are presented as tables and graphs, including the corresponding 95% confidence intervals (95% CI). Statistical analyses were conducted using STATA 15.1 (StataCorp, College Station, TX, USA). 

## 3. Results

### 3.1. Identified Items

Finally, 30 editions were analyzed of all three sports newspapers. Of the 4362 pages reviewed (including supplements), 645 pages—less than 15% of the total—contained some type of health-related material (Table 1). In total, we found 815 health content (HC) units in the sample. MARCA published the major proportion of HC units (*n* = 299, 36.69% of the total) and the greatest number of pages (*n* = 228), but the paper with the highest proportion of pages containing HC was AS (*n* = 225, 15.65%), followed by SPORT (14.46%), and then MARCA (14.29%). 

### 3.2. Descriptive Items

#### 3.2.1. Sections

All of the sports newspapers included specific sections and varied in the coverage of journalistic genres and subgenres. The *Liga BBVA* (Spanish soccer league) sections contained more HC units than any other section (Table 2). After the analyses, it was found that 76.81% of HC units published occupied a space that ranged between 0% and 35% of the page, 11.17% occupied a space between 36% and 70% and, finally, 12.02% occupied a space comprising 71%. Significant differences (*p* < 0.05) were found between the three Spanish sports newspapers analyzed (Table 3). In relation to the journalistic genres, it was found that information (85.77%), opinion (8.96%), and advertising (5.28%) were the most frequent ones (Table 3), although non-significant differences (*p* = 0.192) were found (Table 3). The three best-represented subgenres were news (43.93%), brief articles (18.65%), and full articles/reports (14.11%), and in this case significant differences were found (*p* ≤ 0.001).

#### 3.2.2. Pages

**Figure 1:**
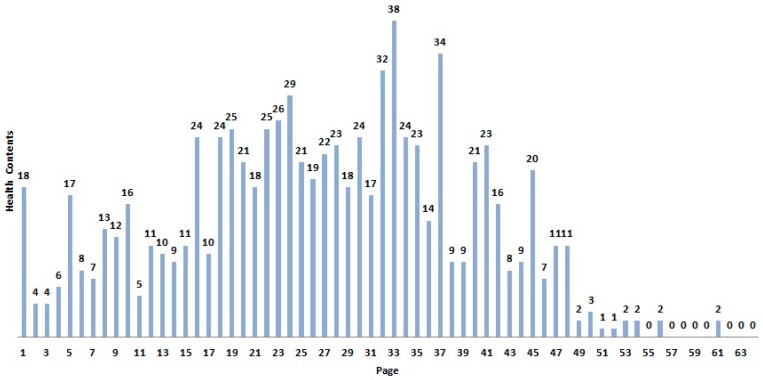
Amount of health content on each page.

The HC appeared highlighted on the frontpage only 69 (8.47%) times (AS: 9.68%; MARCA: 10.37%; SPORT: 4.64%; *p* = 0.041). The HC units were published on almost all pages from the 2nd page (MARCA and SPORT) to the 61st (AS and MARCA). The median and mode were on pages 27 and 34, respectively. This shows that most of the HC units were found on the central pages of each sports newspaper (Figure 1). 

#### 3.2.3. Authors

In relation to authorship, almost all the HC units analyzed (98.77%) were signed by a journalist; this figure was highest for SPORT (100%), followed by AS (98.92%), and finally, MARCA (97.66%). Press agencies, sports organizations, managers, and athletes signed the rest of the content (1.23%). Significant differences were found between the HC units signed by journalists or other collectives (*p* = 0.046).

#### 3.2.4. Health Topics

The three main health topics that were covered most frequently in the three sports newspapers were injuries (52.02%), doping (20.98%), and organic diseases of athletes or their relatives (8.59), and no significant differences were found between the three newspapers studied (*p* = 0.105) (Table 4). 

#### 3.2.5. Illustrations and Sources of Information

**Figure 2:**
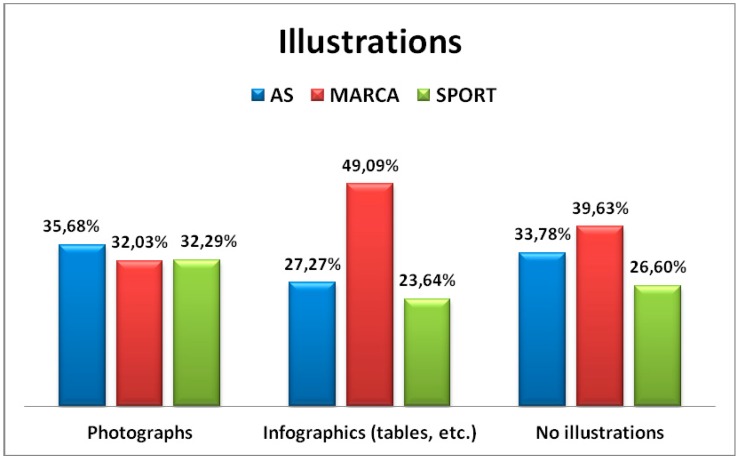
Illustrations included in health content units.

From 815 HC units assessed, 484 included visual materials (59.39%). SPORT was the newspaper including the major percentage of visual materials (57.63%), followed by AS (54.48%), and MARCA (50.17%). From these, 383 units (87.44%) were photographs (Figure 2) and 101 (12.56%) were infographics. No significant differences were found (*p* = 0.061).

The three main sources of information for HC units were press releases (58.10%), public or private sports organizations (17.65%), and interviews and press conferences given by athletes (14.34%). The other sources of information comprised health or sports experts (4.29%) and scientific journals and press agencies (5.64%). No significant differences were found between the newspapers assessed (*p* = 0.151).

### 3.3. Intercoder Reliability

The results for each variable are shown in Table 5. Mean and standard deviation were not calculated because the variables included in this study were categorical. The lowest percent agreement reported was 99.6% in ‘sources of information’ (Scott’s pi (99.4%), Cohen’s kappa (99.4%), and Krippendorff’s alpha (99.4%), while the higher percent agreement was 100% in five variables: ‘pages’, ‘occupied space’, ‘front pages’, ‘authors’, and ‘illustrations’. 

## 4. Discussion

This study has shown that the sample of sports newspapers in Spain regularly published a relevant proportion of health-related material. This study is of particular relevance when taking into account the large number of readers of this specialized press, and since to the best of our knowledge this is the first descriptive analysis covering any health-related material in sports newspapers, either in Spain or in other countries. This descriptive content analysis revealed some key features that may be useful for further analyses. MARCA, which is the most read sports newspaper in Spain [11], published more health-related pages and units. The *Liga BBVA* section was included in all the sports newspapers studied, showing the importance that is given to soccer by the print press. Surprisingly, this section enclosed the largest number of health-related materials. In addition, the main genres and subgenres with health-related material were information and news, and content appeared most frequently on the central pages; topics such as injuries, doping, and organic diseases, being the most frequent. The main source of information was that of press releases, with scientific experts and journals being seldom used.

### 4.1. Identified Items

We surprisingly found more health-related materials in sports newspapers—around 15% of their pages—than in the general newspapers, accounting for between 2% and 7% of all content published [8,36,37,38]. This comparison indicates that the sports print press publishes almost twice as much health-related material as the general press. This, combined with the fact that it is the most read category of print journalism in Spain [11], makes it the best-placed print media for the delivery of health information, at least in relation to the readership impact. In addition, the mean size of HC units per page published in sports newspapers is lower (0%–35%) than those analyzed in the general press (40%–60%) [8,12,32,36,39]. Although the space is minor, the health-related materials are given more visibility in the sports newspapers in terms of the number of pages and HC units published.

The three sports newspapers that were analyzed have a combined daily readership of 2.9 million [11] and this is probably an underestimate, as most subscriptions are held by households, bars, hotels, cafés, working places, etc. [8]. Official statistics [40] from January 2012, the year after the period of analysis, showed that Spain had a population over 14 years old of 39,449,000 inhabitants. This means that at least 8.3% of the Spanish population were exposed to the HC published in sports newspapers, and clearly suggests the potential of sports newspapers as an important tool for delivering health information. Many authors have advised that the publication of health-related materials in the print media may help to promote health and prevent disease, as earlier research suggests that the print media have the power to influence society [41,42]. 

### 4.2. Descriptive Items

#### 4.2.1. Sections

We have observed that health-related material is given certain importance by the sports newspapers. Since the sports newspapers do not have a dedicated health section, such content is placed in other sections. The section containing the greatest number of HC units was Liga BBVA, the section dedicated to soccer. Since this is one of the most popular sports in Spain, health-related materials placed in this section may attain even more visibility. 

#### 4.2.2. Pages

Previous studies [39] have shown that central pages in newspapers are usually considered the most important by the media and this might be a sign of the importance attached by editors to health-related materials. The placement of HC units within the sports newspapers may be an indication of the importance attached to health by editors. The median and mode were on pages 27 and 34, respectively. Aligned with a previous study [39] of health-related material in the mainstream print press, we found that the most popular location was on the central pages of sports newspapers. However, similar studies [8,36] of the mainstream print press have reported that most health-related materials are placed on the first few pages. In this study, 69 HC units were published on the frontpage, meaning that only 8.47% of HC was given this level of importance by editors or by those who decide upon the content of the cover page. This result, although minor, is similar to the findings of previous studies of the general print press [8,36], which reported respectively that 14.3% and 10% of all HC units were published on the frontpage. 

#### 4.2.3. Authors

The results found are similar to previous studies [8,18,36,43] in which the major proportions of HC materials were signed by journalists. In this study, journalists wrote 98.77% of the health content units. Press agencies, sports organizations, managers, and athletes signed the rest of the content. SPORT, published in Barcelona, was signed by journalists in 100% of cases.

#### 4.2.4. Health Topics

Regarding health topics, it is necessary to mention the important influence the media has on the health topics that are selected every day for publication. In fact, everything that appears in a newspaper has a certain priority, which has been previously decided by the editorial team. During our sampling period, the health topics most frequently covered in the sports newspapers were injuries, doping, and diseases of athletes or their relatives. Joint injuries (shoulder, elbow, wrist, hip, knee, foot, etc.) and muscular injuries (pubis, cufflinks, etc.) were the main topics. This finding is consistent with an earlier study of the UK sports press which also found that sports newspapers provide detailed descriptions of athletes’ health issues, with soccer-related stories dominating [18]. The next most frequently reported issue was doping, which accounted for 20.98% of HC and included stories mostly on Marta Domínguez (athletics), Alberto Contador, and Alejandro Valverde (cycling). The deaths of athletes and/or their relatives, tributes, etc., and diseases of athletes and their relatives (influenza, mumps, gastroenteritis, E. coli, ulcerative colitis, etc.) were other themes, e.g., Gustavo’s (son of Martins, a player of Granada F.C.) leukemia and Tito Vilanova’s (Barcelona F.C. trainer) parotid gland tumor. Stories about mental health issues (mental fatigue, alcoholism, depression, etc.) and disability issues (blindness, adapted sport, deafness, etc.) were also discussed. Attacks on athletes, athletes’ relatives, referees, and fans were less frequent health-related topics. These findings are in line with those of two previous studies [8,36], which also reported that public health issues received more coverage than diseases. However, it contrasts with another study that reported greater coverage of medical diseases [12].

#### 4.2.5. Sources of Information

With regard to sources of information, we should first take into account that most health stories in newspapers are written by non-specialist health journalists [8,31,42,43]. As with this study, we also found that the main sources of information for health stories were press releases. This finding contrasts with two previous studies wherein the main sources of information were obtained from politicians/governmental institutions [8,42].

#### 4.2.6. Study Limitations

Although our results are of great relevance, especially to health research and the media, the limitations of the study should be taken into account. Despite using a wide sampling period (30 editions of each sports newspaper), the first limitation is based on the sampling period, December 2011; we cannot generalize these findings to other periods of time as these may differ. As a result of the variety and diversity of media performance, reflecting different opinions, catering for different needs, offering choices, etc. [44], the findings in our specific sample and time period should be considered as matchless as they cannot be replicable in other media analyses or periods of time. This is due to the variety of news that is published daily by sports-specialized newspapers which is determined by many factors, even influencing the readers according to the theory of framing [45]. This means that different presentations of sports newspaper articles can alter the reader’s interpretations causing a variety of effects. Moreover, by exploring the months and year of our analysis specifically, we can see that December is a month where there is less sports events than other months because of the Christmas and New Years’ eve holidays. Although sports newspapers keep publishing during this period, we must acknowledge that the content routine may be affected by this special situation. If we look to the year 2011 and particularly in December, we found some specific news related to different health topics, but the following were particularly highlighted: (1) Doping in Spanish sport: the case of the Spanish cyclist Alberto Contador (winner of the Giro d’Italia 2008 and 2015, Tour of France 2007 and 2009, and Vuelta España 2008, 2012 and 2014); (2) Son of the Portuguese international soccer player Carlos Martins suffers from a rare disease; (3) Tito Vilanova (coach of F.C. Barcelona) suffers cancer; (4) The Spanish tennis player Rafael Nadal (winner of 17 Grand Slam tournaments) suffers from health problems (knee and shoulder); (5) Physical and mental fatigue of soccer players due to the significant number of matches played both in the Spanish league and international competitions. However, there are several similarities between our findings and those of similar studies [8,12,18,31], which may suggest the validation of the results.

The second limitation is that we only analyzed printed sports newspapers, although the exponential growth of the Internet is moving readers from print editions to online versions. The new online modality has the capacity to reach a truly global audience and this, together with the fact that a large proportion of online newspapers (at least in the sports press) is free-access, means that it is crucial to consider their content as well. Because our analysis did not extend to the online versions, our results can only be applied to the print media. Finally, this study was limited to a descriptive analysis of the health-related materials in sports newspapers. We did not analyze trends, ideologies, or the rigor of the information published elsewhere; the main findings of this study were compared with similar studies carried out with mainstream newspapers because there were no previous studies analyzing sports newspapers specifically. Therefore, we would like to encourage deeper analysis of health-related material in the sports media. Future studies in this field should: (1) Include a longer study period; (2) Compare newspapers in different countries; (3) Compare mainstream media with sports newspapers; and (4) Assess different periods of time. This descriptive analysis should be considered as the beginning of a research field.

## 5. Conclusions

This study is the first to assess health-related materials in sports newspapers using a comprehensive content analysis. Such content was found to be frequently reported throughout the studied period in the Spanish sports newspapers selected, suggesting that this might be one of the most important sources of health content for Spanish readers. Sports newspapers publish more health-related material than the mainstream print press. Sports injuries were the most frequently covered health issue and soccer was the dominant section. Finally, press releases were the main source of information for health-related materials. In addition to considering the great influence of sports newspapers as a source of information on issues such as injuries, doping, and organic diseases in athletes and their families, this type of press also seeks to promote a healthy lifestyle and policies with the ultimate goal of contributing to improving quality of life of the population (for example, through the development of campaigns to prevent sexually transmitted diseases, healthy eating, level control of cholesterol/hypertension/diabetes, prevention of traffic accidents, etc.). Another important task of this specialized media is responsible health education avoiding sensationalism.

## Figures and Tables

**Table 1:** Sample information.

Newspaper	Pages Reviewed *n* (%)	Pages Published with HC *n* (%)	HC Units *n* (%)
AS	1438 (32.97)	225 (15.65)	279 (34.23)
MARCA	1596 (36.59)	228 (14.29)	299 (36.69)
SPORT	1328 (30.44)	192 (14.46)	237 (29.08)
TOTAL	4362 (100)	645 (14.79)	815 (100)

HC: Health content.

**Table 2:** Main sections with health content.

AS		MARCA		SPORT		ALL	
Sections	*n* (%)	Sections	*n* (%)	Sections	*n* (%)	*n* (%)	*p*-Value
Liga BBVA	116 (41.58)	Liga BBVA	137 (45.82)	Liga BBVA	85 (35.86)	338 (41.47)	0.012
More sport	66 (23.66)	Polideportivo	54 (18.06)	Polideportivo	76 (32.07)	196 (24.05)	
International	32 (11.47)	International	27 (9.03)	Soccer Planet	28 (11.81)	87 (10.67)	
Liga Adelante	15 (5.38)	Liga Adelante	16 (5.35)	Liga Adelante	6 (2.53)	37 (4.54)	
Others ^1^	50 (17.92)	Others ^2^	65 (21.74)	Others ^3^	42 (17.72)	257 (19.26)	
Total	279 (100)	---	299 (100)	---	237 (100)	815 (100)	

^1^ Theme of the day, letters to the director, advertising, supplements, free throws (basketball), etc. ^2^ MARCA says, supplements, weekly recommendations, advertising, MARCA survey, letters to the director, etc. ^3^ Advertising, confidential, basketball, etc.

**Table 3:** Space occupied, genre and subgenre included in every health content assessed.

		AS	MARCA	SPORT	TOTAL	
		*n* (%)	*n* (%)	*n* (%)	*n* (%)	*p*-Value
Occupied space	0%–35%	227 (81.36)	213 (71.24)	186 (78.48)	626 (76.81)	< 0.05
	36%–70%	24 (8.60)	33 (11.04)	34 (14.35)	91 (11.17)	
	71%–100%	28 (10.04)	53 (17.72)	17 (7.17)	98 (12.02)	
	Total	279 (100)	299 (100)	237 (100)	815 (100)	
Genre	Advertising	18 (6.45)	14 (4.68)	11 (4.64)	43 (5.28)	0.192
	Information	228 (81.72)	261 (87.29)	210 (88.61)	699 (85.77)	
	Opinion	33 (11.83)	24 (8.03)	16 (6.75)	73 (8.95)	
	Total	279 (100)	299 (100)	237 (100)	815 (100)	
Subgenre	News	91 (32.62)	138 (46.16)	129 (54.43)	358 (43.93)	< 0.001
	Article	25 (8.96)	52 (17.39)	38 (16.03)	115 (14.11)	
	Brief article	96 (34.41)	21 (7.02)	35 (14.77)	152 (18.65)	
	Others *	67 (24.01)	88 (29.43)	35 (14.77)	190 (23.31)	
	Total	279 (100)	299 (100)	237 (100)	815 (100)	

* Interviews, advertising, editorials, reports, columns, chronicles, commentaries, obituaries, letters, surveys.

**Table 4:** Health topics.

	AS	MARCA	SPORT	TOTAL	
	*n* (%)	*n* (%)	*n* (%)	*n* (%)	*p*-Value
**Injuries**	150 (53.76)	167 (55.85)	107 (45.15)	424 (52.02)	0.105
**Doping**	63 (22.58)	55 (18.39)	53 (22.36)	171 (20.98)	
**Organic diseases**	18 (6.45)	29 (9.70)	23 (9.70)	70 (8.59)	
Others *	48 (17.20)	48 (16.05)	54 (22.78)	150 (18.41)	
**Total**	279 (100)	299 (100)	237 (100)	815 (100)	

* Others: advertising, necrological, mental health, aggressions, disability.

**Table 5:** Intercoder reliability and percentages.

Variable	Percent Agreement (*n*)	Scott’s Pi	Cohen’s Kappa	Krippendorff’s Alpha (Nominal)	Agreements (*n*)	Disagreements (*n*)	Cases (*n*)	Decisions (*n*)
Pages (cols 1 & 2)	100	1	1	1	815	0	815	1630
Sections (cols 1 & 2)	99.8	0.997	0.997	0.997	813	2	815	1630
Genre (cols 1 & 2)	99.9	0.995	0.995	0.995	814	1	815	1630
Subgenre (cols 1 & 2)	99.9	0.998	0.998	0.998	814	1	815	1630
Occupied space (cols 1 & 2)	100	1	1	1	815	0	815	1630
Frontpages (cols 1 & 2)	100	1	1	1	815	0	815	1630
Authors (cols 1 & 2)	100	1	1	1	815	0	815	1630
Health topics (cols 1 & 2)	99.9	0.998	0.998	0.998	814	1	815	1630
Illustration (cols 1 & 2)	100	1	1	1	815	0	815	1630
Sources of information (cols 1 & 2)	99.6	0.994	0.994	0.994	812	3	815	1630
